# Issues associated with assessing nuclear localization of N-terminally unphosphorylated β-catenin with monoclonal antibody 8E7

**DOI:** 10.1186/1745-6150-4-5

**Published:** 2009-02-02

**Authors:** Meghan T Maher, Annette S Flozak, Alyssa M Hartsell, Susan Russell, Rohinee Beri, Ofra N Peled, Cara J Gottardi

**Affiliations:** 1 Department of Medicine, Northwestern University Feinberg School of Medicine, Chicago, IL 60611, USA; 2 The Integrated Graduate Program in the Life Sciences, Northwestern University Feinberg School of Medicine, Chicago, IL 60611, USA; 3 Robert Lurie Cancer Center, Northwestern University Feinberg School of Medicine, Chicago, IL 60611, USA; 4 Department of Natural Sciences, National-Louis University, Chicago, IL 60603, USA

## Abstract

**Background:**

β-catenin is a dual function adhesion/transcriptional co-activator protein, and both functions are critical for normal tissue homeostasis. Since the transcriptional functions of β-catenin are more often implicated in various disease processes, there is much interest in the development and use of reagents to interrogate spatial and temporal evidence of β-catenin nuclear signaling in cells and tissues. An important study demonstrated that the signaling form of β-catenin is specifically unphosphorylated at residues S37 and T41, and suggested that this form exhibits a propensity for cytosolic/nuclear accumulation relative to the total pool of β-catenin.

**Results:**

We show that monoclonal antibody, 8E7, which recognizes the signaling form of β-catenin specifically unphosphorylated at S37 and T41 (Active B-Catenin, ABC), also cross-reacts with a widely expressed, variably accessible nuclear antigen that is not β-catenin. In cell types commonly used to study Wnt activation, this non-specific nuclear staining can be robust, obscuring the ABC signal. Definitive detection of nuclear localized ABC can be confirmed through an ability of classical cadherins to sequester ABC to cell junctions. In tissues, milder antigen retrieval methods can reduce the accessibility of mAb 8E7 to this cross-reacting nuclear antigen.

**Conclusion:**

These findings reveal that interpretation of nuclear, signaling active β-catenin using monoclonal antibody 8E7 should be considered judiciously, and in conjunction with independent methods.

**Reviewers:**

This article was reviewed by Frank J. T. Staal (nominated by Rachel Gerstein), Jyoti M. Sen (nominated by Avinash Bhandoola) and Manabu Sugai.

## Background

β-catenin is a professional binding protein, whose function is largely dictated by its particular partner. When β-catenin interacts with cadherin adhesion receptors, it serves to critically link these receptors to the cytoskeleton (reviewed in [1]). In the nucleus, β-catenin partners with LEF/TCF-family DNA-binding proteins, forming an essential link between their DNA-binding function and the recruitment of factors required for chromatin remodeling and transcriptional activation (reviewed in [2]). In most cell types, the adhesive function of β-catenin predominates, due to the constant synthesis of cadherin/catenin complexes during steady-state conditions [3]. During tissue development and repair, a cadherin-free, cytosolic form of β-catenin is generated by extracellular Wnt ligands. These Wnts engage cell surface receptors to initiate a signal transduction pathway that largely serves to promote the post-transcriptional stabilization and nuclear localization β-catenin [4,5]. Recruitment of β-catenin to LEF/TCF-bound promoters ultimately leads to the activation of genes required for distinct cellular outcomes [6].

While cytosolic stabilization of β-catenin has long been considered a hallmark of Wnt-activation, it is now appreciated that β-catenin which remains hypophosphorylated within the GSK3β-consensus region constitutes the signaling form [7,8]. Strong evidence for this model has relied on the generation of a monoclonal antibody (mAb), which was screened to recognize a peptide corresponding to β-catenin (amino acid residues 36–44), specifically when T41 and S37 are *not *phosphorylated (8E7, Upstate Biotechnology/Millipore [9]). This antibody recognizes the signaling Active form of β-Catenin, or ABC [8]. Since this reagent allows investigators to examine changes in β-catenin N-terminal modification using simple immuno-detection methods, it has become a popular tool to begin explorations into whether a cell has been the recipient of a Wnt or Wnt-like activity. A similarly named monoclonal antibody, 8E4, is incorrectly marketed as an antibody that also recognizes β-catenin "non-phosphorylated" at the N-terminal GSK sites, and has recently been shown to recognize β-catenin at a completely different epitope [10].

As part of our own efforts to understand how phosphorylation of β-catenin's N-terminus alters its nuclear signaling activities, we discovered that while mAb 8E7 indeed recognizes cytoplasmic/nuclear ABC, this antibody also cross-reacts with a nuclear antigen in a number of cell types. Because nuclear staining persists in a cell line where the β-catenin gene is deleted by homologous recombination [11], we know that this nuclear antigen is not β-catenin. This study offers two ways to improve the reliable use of this antibody. First, cadherin overexpression analysis can be used to deplete a nuclear signal that is due to ABC. Second, milder antigen retrieval methods appear to reduce the accessibility of mAb 8E7 to this cross-reacting nuclear antigen.

## Results and Discussion

**Figure 1:**
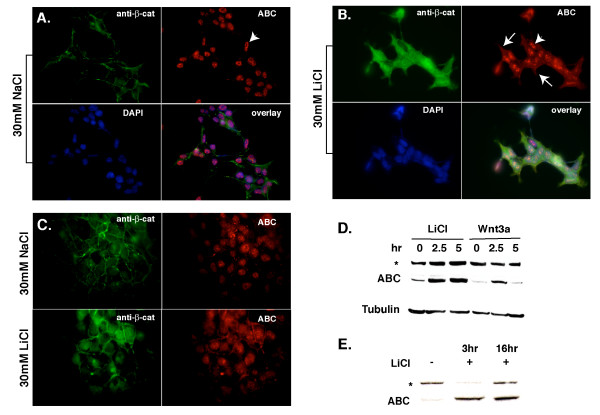
**Subcellular localization of signaling and total forms of β-catenin in HEK 293T cells**. **A-C**: HEK 293T cells were treated with 30 mM NaCl (control, **A**) or 30 mM LiCl (B) for 16 hours to induce the post-transcriptional accumulation of cytosolic/nuclear β-catenin. Cells were co-immunostained using a polyclonal antibody raised against the consensus GSK3 phosphorylation sites of human β-catenin (#06-734; Millipore), and a monoclonal antibody that recognizes ABC (8E7, Millipore). DAPI was used to stain cell nuclei. **A**. NaCl (control)-treated cells display a small amount of junctional and cytoplasmic β-catenin (green), while ABC (red) is found exclusively in the nuclear compartment. **B**. In the presence of Wnt signaling, cells show increased expression and colocalization of total-β-catenin and ABC in the cytoplasm and nucleus. Note that the punctate, nuclear ABC staining remains largely unchanged after LiCl induction (arrowheads). Rather, a diffuse cyto/nucleoplasmic ABC staining pattern is most evident (arrows in B). No staining is observed with goat-anti-rabbit IgG (GAR) and goat anti-mouse IgG (GAM) secondary antibodies alone (not shown). Exposure times for anti-β-catenin (green) and ABC (red) images are 1215 and 1632 milliseconds, respectively. **C**. Same experiment as A&B except exposure times were matched for the two β-catenin antibodies (1000 ms). **D&E**. Immunoblot analysis of HEK cells treated with LiCl or recombinant mouse Wnt3a protein (R&D Systems #1324-WN/CF). Note that 8E7 detects ABC as well as a non-specific band at ~160 kDa (*).

Through our efforts to understand how the N-terminally, hypophosphorylated form of β-catenin is regulated, particularly in the context of fibrotic disorders where this pathway has been recently shown to play a causal role [12], we discovered that primary lung fibroblasts exhibited strong nuclear staining using the ABC antibody (data not shown). This either suggested that our fibroblast cultures were in a state of constitutive, Wnt/β-catenin signaling activation or, alternately, raised questions about the specificity of mAb 8E7 in these cells. To address this, we subjected HEK293T cells to Wnt pathway activation using lithium chloride as previously described [8], followed by immunofluorescence double-labeling with the antibody that specifically recognizes β-catenin that remains unphosphorylated at S37 and T41 (mAb 8E7), and an antibody that presumably recognizes all forms of β-catenin (#06-734; Millipore). While lithium chloride clearly increased the nuclear/cytoplasmic staining observed with both antibodies, substantial nuclear staining using the ABC antibody was observed in uninduced HEK293T cells (Figure 1A–C). Short-term exposure of HEK cells with recombinant Wnt3a protein induced ABC protein levels comparable to LiCl-treatment (Figure 1D&1E). No obvious increase in nuclear ABC was observed with after incubation with recombinant Wnt3a protein, or transient transfection with a Wnt3a encoding plasmid (not shown). Altogether, these results show that while mAb 8E7 readily recognizes ABC by immunoblot analysis, the high level of background nuclear staining obscures the detection of ABC by immunofluorescence analysis.

**Figure 2:**
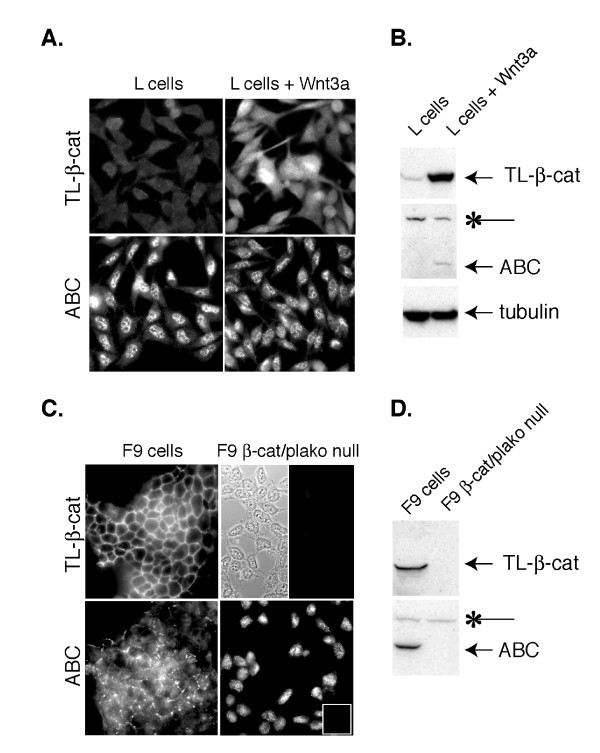
**Nuclear staining associated with mAb 8E7 is observed in β-catenin null cells**. **A&B**: Mouse L cell fibroblasts +/- Wnt3a **A**. Immunofluorescence staining of total (TL-) β-catenin (mAb clone 14, BD Biosciences) and ABC in mouse L cells stably expressing Wnt3a (right) or an empty vector (left). Note prominent nuclear staining in both Wnt3a expressing and non-expressing cells using 8E7 mAb. Exposure times for TL-β-catenin and ABC images are 1313 and 1439 milliseconds, respectively.**B**. Corresponding immuno-blot. Note that 8E7 detects non-specific band at ~160 kDa (*). **C&D**: WT and β-catenin/plakoglobin null F9 teratocarcinoma cells immunostained with antibodies that recognize TL- and ABC. **C**. The split panel (upper right) shows the presence of β-catenin/plakoglobin null F9 cells on the coverslip (phase) that are not stained with the TL-β-catenin antibody, but show a diffuse cytoplasmic/nuclear stain with mAb 8E7 (lower right). Inset shows the GAM secondary antibody control. Exposure times for TL-β-catenin and ABC images are 228 and 453 milliseconds, respectively. **D**. Corresponding immunoblot shows that 8E7 detects a non-specific band at ~160 kDa in F9 cells (*). It is unclear whether this non-specific band contributes to background nuclear staining.

To interrogate the specificity of mAb 8E7, we subjected two cell lines that express no β-catenin protein to immunofluorescence and Western blotting analysis (Figure 2). Mouse L cells are a fibroblastic line that produces little β-catenin protein due to an absence of cadherin expression and constitutive turnover of newly synthesized β-catenin [3,13,14]. While the stable transfection of Wnt3a plasmid results in the upregulation of both total and hypophosphorylated forms of β-catenin by immunoblotting, confirming that these cells can respond to a Wnt signal appropriately (Figure 2B), there is a strong nuclear staining pattern detected by 8E7 in control L cells that remains unaltered by the presence of Wnt3a (Figure 2A). These data suggest that the nuclear staining observed with 8E7 may not be due to β-catenin. To address this more definitively, we used a cell line where both β-catenin, and the related plakoglobin, genes are deleted through homologous recombination [11]. While β-catenin/plakoglobin null F9 cells exhibit no β-catenin protein by immunoblot analysis (Figure 2D), a diffuse nuclear staining pattern remains when these cells are stained with mAb 8E7. Together, these data demonstrate that 8E7 cross-reacts with a nuclear antigen that is not β-catenin.

**Figure 3:**
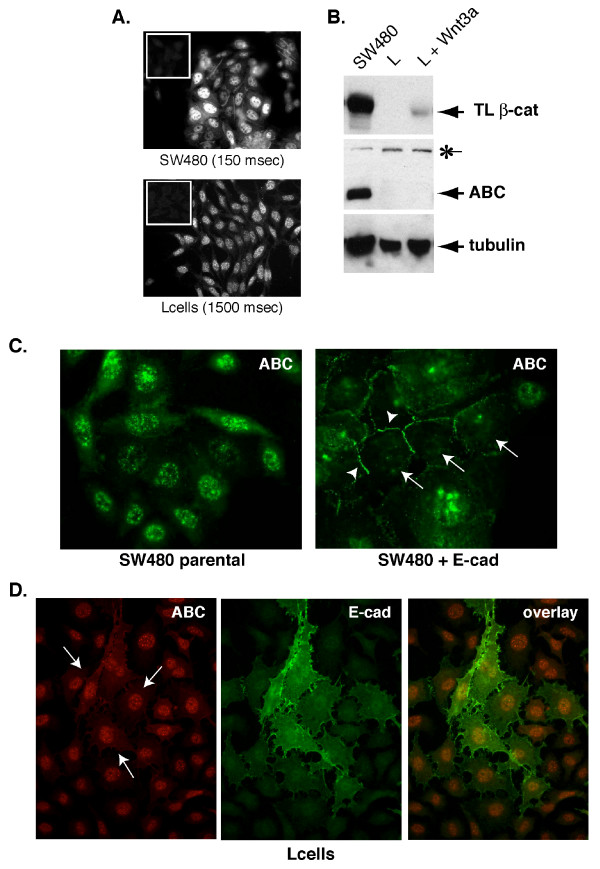
**Assessment of nuclear localized ABC can be verified by cadherin-mediated depletion of the nuclear signal**. **A&B**. **A**. Immunofluorescence staining of ABC with mAb 8E7 in SW480 human colon carcinoma and mouse L cells. Note that staining is qualitatively similar, but the exposure time for SW480 cells is one tenth that of the L cells. Insets (upper left) show secondary alone controls. **B**. Western analysis confirms that ABC is significantly more abundant in SW480 cells than L cells (50 μg protein/lane). Non-specific, ~160 kDa band recognized by 8E7 is indicated by (*). **C**. ABC staining in SW480 cells stably expressing E-cadherin (SW480 + E-cad; [19]) and parentals. Note that E-cadherin expression increases junctional staining (arrowheads) and depletes nuclear staining (arrows) of ABC. Exposure times for parental and cadherin-expressing images are 276 and 313 milliseconds, respectively. **D**. Double-immunolabeling of ABC (red; 1300 ms) in mouse L cells transiently transfected with E-cadherin (green; 984 ms). Note that in contrast with SW480s, nuclear staining in L cells cannot be depleted by cadherin expression (arrows).

While 8E7 non-specifically detects nuclei, this signal is relatively weak compared with cell lines that show strong activation of β-catenin signaling, such as the widely used human colon cancer cell line, SW480 (Figure 3A&3B). To prove that the nuclear signal observed in SW480 cells is indeed due to β-catenin, we stably reintroduced E-cadherin into these cells and observed a nearly complete depletion of the nuclear ABC signal, and recruitment to cell-cell junctions (Figure 3C). Redistribution of the ABC signal was not observed when E-cadherin was expressed in mouse L cells (Figure 3D), consistent with data showing that this nuclear signal is not due to β-catenin (Figure 2A&2B). Since the β-catenin specific and non-specific staining patterns observed with 8E7 are qualitatively indistinguishable, definitive detection of nuclear localized ABC can be confirmed through an ability of classical cadherins to sequester this form of β-catenin to cell junctions.

**Figure 4:**
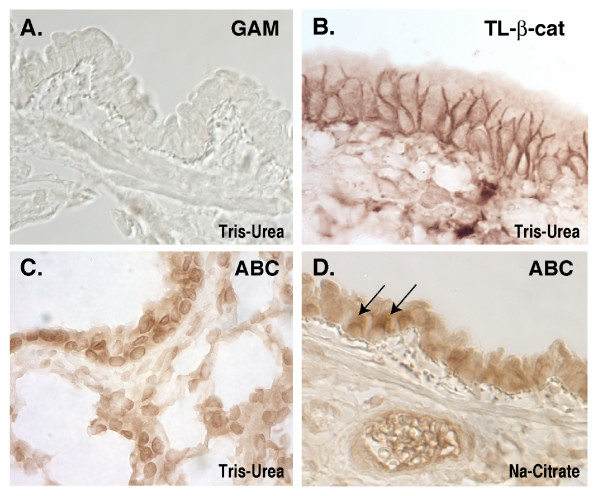
**Detection of ABC depends on antigen retrieval method**. **A-D **Rat lung paraffin sections. Sections treated with boiling Tris-Urea (**A-C**) or sodium citrate buffer (**D**) before incubation with the following antibodies: (**A**) GAM-HRP secondary alone, (**B**) anti-TL-β-catenin, (**C&D**) anti-ABC (8E7). Note that sections treated with Tris-Urea buffer unmask an epitope detected by ABC and present in all nuclei (**C**). Sections treated with sodium citrate buffer show diminished nuclear background, and instead reveal a sub-population of lung airway epithelial cells that contain the ABC epitope (arrows; **D**).

The original description of mAb 8E7 showed that this antibody could detect the signaling form of β-catenin in developing intestinal crypts by standard immunohistochemical analysis [9]. More recently, this antibody has been used in adult liver, where it revealed a gradient of ABC staining along the perivenous/periportal axis to control the expression of genes required for ammonia detoxification [15]. In other studies, nuclear staining with mAb 8E7 was observed after genetic loss of components that inhibit β-catenin signaling [16,17]. Through our own efforts to use this antibody to identify epithelial cell types maintained by β-catenin signaling in adult lung, we found that mAb 8E7 could detect all nuclei within the section (Figure 4C). In striking contrast, this non-specific nuclear staining pattern was not observed when sections were treated with milder antigen retrieval methods, such as boiling citrate buffer (Figure 4D). Instead, these latter conditions revealed a diffuse cytoplasmic/nuclear staining within a subpopulation of cells, similar to what was observed in intestinal crypts [9,18]. The staining in Figure 4D is also consistent with evidence that the lung airway epithelium contains progenitors that can undergo renewal after injury [18], as well as our own data showing that these lung airway cells are sensitive to Wnt/β-catenin pathway activation using the Axin2-^LacZ/+ ^reporter mouse (Flozak and Lam* et al., unpublished). Taken altogether, the variable accessibility of this cross-reacting nuclear antigen may explain differences in detecting a specific nuclear ABC signal over this background antigen (e.g., Figure 1 versus (1)).

## Conclusion

We show that mAb 8E7, in addition to specifically recognizing an N-terminally hypophosphorylated form of β-catenin as previously demonstrated [8,9], also cross-reacts with a variably accessible nuclear antigen that is not β-catenin. These findings demonstrate that the ability of 8E7 to accurately capture evidence of Wnt signaling in cells or tissues critically depends on the strength and/or duration of the Wnt signal, relative to the accessibility of this background antigen. In cell cultures, nuclear accumulation of ABC can be easily verified by cadherin over-expression and redistribution of ABC to cell-cell junctions. In tissue sections, the non-specific nuclear staining can be reduced by use of gentler antigen retrieval methods. Altogether, our findings underscore the obvious need to evaluate β-catenin signaling activation through multiple independent assays, such as *in vitro *and *in vivo *reporters, upregulation of the more "universal" target genes (e.g., Axin2 and others; see the Wnt Homepage at ), in addition to providing evidence for increases in cytosolic and N-terminally unphosphorylated β-catenin by immunoblot analysis.

## Methods

### Immunofluorescence staining

300,000 HEK293T, L cells or L cells stably expressing Wnt 3a were seeded onto sterile 22 × 22 mm glass coverslips within a 35 mm Petri dish in DMEM-10%FBS and cultured for 48 hours. Media was then aspirated and slips were washed three times with PBS containing calcium and magnesium (PBS^++^). Cells were fixed in ice-cold anhydrous methanol for 10 minutes followed by three washes with PBS^++^. Cells were permeabilized and blocked for 30 minutes in PBS with 0.3%Triton-X100 and 0.3%BSA. Monoclonal mouse anti-activated β-catenin (Upstate clone 8E7, lot# 32519, 0.39 mg/ml, and #31776, 0.47 mg/ml; diluted to a working concentration of 1–2 μg/ml) and monoclonal mouse anti-β-catenin (0.25 μg/ml; BD Transduction, clone 14; Upstate, #06-734 at 1 μg/ml) were diluted in PBS with 0.3%Triton-X100, 0.3%BSA and 5% normal goat serum (NGS) and centrifuged briefly before use. Slips were incubated with primary antibody for 60 min at room temperature, followed by washing 10 dips each in 300 ml of PBS with 0.3%Triton-X100 and PBS alone. Cells were then incubated with a 1:500 dilution of goat anti-mouse or rabbit IgG-conjugated Alexa Fluor 488 or 568 (Molecular Probes; 0.25 μg/ml) in PBS with 0.3%Triton-X100, 0.3%BSA and 5% NGS centrifuged briefly before use for 60 min at room temperature. Slips were washed 10 dips each in a large volume of PBS with 0.3%Triton-X100, PBS alone, and finally distilled water. Coverslips were mounted under Gelvatol containing 0.1% DABCO and air dried one hour before storage at -20°C until viewing. Slides were viewed and imaged with a Zeiss Axioplan epifluorescence microscope equipped with a 63× objective.

### Immunohistochemistry

Immunoperoxidase experiments were performed on 3% phosphate-buffered, formalin-fixed, paraffin-embedded lung tissue according to standard protocols. For antigen unmasking, tissue sections were softly boiled in 0.5 M Tris-HCl, pH9.5 with 5% urea, or 10 mM citrate buffer for 6 minutes. Signal amplification was carried out using the streptavidin/biotin ABC system (Vector Labs).

### SDS-PAGE and Immunoblotting

Cells were lysed in standard RIPA buffer (1%Triton X-100, 0.1%SDS, 150 mMNaCl, 20 mM Tris pH 7.5, 5 mM EDTA) containing protease and phosphatase inhibitors (Roche). Protein content was assayed using the Bradford method (Bio-Rad). Proteins were separated by SDS-PAGE and electrophoretically transferred to BioTrace NT nitrocellulose (Pall). Membranes were stained with Ponceau S solution to evaluate protein transfer. Membranes were blocked in 5% milk-TBS-Tween and incubated with primary and secondary antibodies diluted in 5% milk-Tris-Buffered Saline-Tween. The ABC antibody was used at 0.2 μg/ml; anti-β-catenin clone 14 (BD Transduction) was used at 0.25 μg/ml. Immunoblots were developed in ECL solution (GE Amersham) and exposed to chemiluminescent-compatible film (Hyperfilm-ECL, GE Amersham).

## Abbreviations

TCF: (T-cell factor); LEF: (Lymphocyte Enhancer Factor); ABC: (Active β-Catein); mAb: (monoclonal antibody).

## Competing interests

The authors declare that they have no competing interests.

## Authors' contributions

MTM, ASF and AMH carried out immuno-fluorescence and -blotting experiments; SR, RB and ONP performed immunohistochemistry experiments; MTM, ASF and CJG wrote the manuscript. All authors read and approved the final draft.

## Reviewer's comments

### Reviewer 1

Jyoti Misra Sen

Lymphocyte Development Unit, Laboratory of Immunology, NIH, National Institute on Aging. Baltimore, MD

#### Comment

In this study Maher et al. evaluate the specificity of an important antibody reagent 8E7, which was believed to identify unphosphorylated beta-catenin, generally believed to be the signaling form of the protein. Their analysis clearly demonstrates that the 8E7 antibody cross-reacts with a non-specific protein antigen in cells that lack beta-catenin expression.

Specifically data shown in figure 2A, demonstrate that L cells express low levels of beta-catenin which is dramatically increased upon treatment with Wnt-3a. However, staining with 8E7 remains high with or without Wnt-3a expression. These data are further supported with similar observations in F9 cells in which expression of beta- and gamma-catenin is deleted by homologous recombination. I agree with the conclusion of the authors that this antibody should be used with caution, especially for immunohistochemical analysis.

### Reviewer 2

Frank J.T. Staal

Erasmus MC, Rotterdam, The Netherlands

#### Reviewer Comment #1

The 8E7 antibody, developed in the laboratory of Dr. Hans Clevers recognizes the dephosphorylated N-terminus of β-catenin, the signalling competent form of β-catenin. This reagent was developed in order to better follow the fate of canonical Wnt signalling at the protein level, rather than simply assaying for increased levels of total or nuclear β-catenin which can occur independently of active Wnt signalling (as for instance elegantly shown by M. Watermanís lab). The development of these types of reagents was first described by Mascha van Noort et al in JBC and further on one of the developed antibodies, 8E7 or anti-ABC (activated-beta-catenin) was shown to report active Wnt signalling and (Staal et al, EMBO REP), but not increases in β-catenin by other means. It has been described before in the JBC paper that 8E7 also recognizes a ubiquitous, apparently non-Wnt regulated protein of higher MW (Fig 3 of that paper), but for most applications that has not been a problem, certainly in protein blots, but also in immuno fluorescence applications when done as originally described.

Cara Gottardi and co-workers now describe in a series of carefully conducted experiments that other antigen retrieval methods would lead to detection of this ubiquitous protein, which could confuse researchers and therefore is a worthwhile caveat to report. The authors should mention the main confusion surrounding this antibody has been the inappropriate marketing of a pan-β-catenin antibody advertised as also recognizing dephosphorylated β-catenin (which it does not do) with the similar name of 8E4. Despite a clarification we published last year in Blood, the incorrect antibody still appears every now and then in the literature. Besides that, 8E7 recognizes this "variably accessible" nuclear antigen that is not β-catenin, but it also correctly recognizes dephosphorylated β-catenin, and thus far is still the best reagent we have.

#### Our response

Frank Staal raises an important issue that merits clarification. Some anti-β-catenin antibodies are currently marketed by companies as "non-phospho" or "de-phospho" β-catenin (e.g., mAb 8E4, #05-601 and polyclonal Ab #06-734, respectively; Millipore). With regards to 8E4, a recent clarification published by the Staal group showed that 8E4 recognizes an epitope that lies well outside the GSK3 regulatory region [10]. This clarification is yet to be incorporated into the company's data sheet for 8E4. With regards to Ab #06-734, although this antibody was *raised against *a peptide antigen corresponding to β-catenin's unphosphorylated GSK3 sites, this antibody should not be confused with an antibody like 8E7, which was *specifically screened to only recognize *β-catenin proteins that are unphosphorylated at certain GSK3 residues. In other words, the "non-phospho" status of an antigen does not ensure that the antibodies that recognize this region will only recognize the non-phosphorylated protein. Rather, a rigorous screening method (as part of the selection of 8E7, [9]) is required to ensure specific recognition of unphosphorylated epitopes.

#### Reviewer comment #2

That a similar epitope is recognized by an antibody can be a problem under conditions with low active Wnt signals, is obvious. The notion that there is some background staining in unstimulated 293T cells, was reported previously (Staal et al, EMBO REP) and with strong activation signals a clear increase is seen. I think it would be helpful if authors would include an experiment with milder Wnt stimulation, for instance by transfecting Wnt1 into 293T cells.

#### Our response

We concur with the reviewer's statement that all antibodies cross-react with epitopes from other proteins, and that it is the responsibility of the investigator to understand the specific "signal-to-noise" ratio in their particular system. With regards to the non-specific nuclear staining associated with 8E7, titrating the antibody to reduce this background (reducing "noise"), or robustly activating the pathway (increasing "signal"), can certainly help. But not all cell systems can be manipulated this way. In this regard, Staal et al. nicely demonstrate that activation of HEK cells with Wnt-1 revealed enhanced nuclear staining with mAb 8E7 over the uninduced condition [9]. However, we could not see this enhanced nuclear accumulation of ABC in HEK cells treated with LiCl or Wnt3a (Figure 1). It is possible that our laboratories are working with different subclones of HEK293 cells that exhibit different sensitivities to Wnt pathway activation. Alternatively, the accessibility of the cross-reacting nuclear antigen may depend on cell plating, fixation and permeability conditions.

#### Reviewer comment #3

I fully agree that besides antibody staining other methods to measure Wnt activity should be used, but a cautionary note should be added that even the so-called universal Wnt target genes (such as Axin2) may a) be regulated by other signals as well b) not always show up in all tissues as target genes (as is our experience). If authors can correct these points I see no reasons to object to publishing a cautionary report that when inappropriately used or with low Wnt signals, care should be taken one is actually measuring canonical Wnt signalling.

#### Our response

While negative feedback regulators of Wnt pathway activation like Axin2, naked and DKK  tend to be more generally expressed across different cell types, we acknowledge the reviewer's point that there may be no "universal" Wnt pathway target gene(s). Thus, Wnt-activated targets for a particular cell and tissue type must be determined on a case-by-case basis.

### Reviewer 3

Manabu Sugai

Kyoto University, Center For Genomic Medicine, Graduate School of Medicine

### General comments

In this study, the authors have shown that monoclonal antibody 8E7 recognizes not only β-catenin but also other nuclear molecule(s) in some cell lines. From these observations, they recommend milder antigen retrieval method for tissue section staining using this antibody. However, this condition is very similar as described in Dr. Clevers's lab (J Biol Chem 277, 17901). There are some novel findings that are of interest, but the significance of the findings is not clear and the proposed procedures in using this antibody are not so informative comparing to the previous one.

To determine the significance of weak signals appeared in some staining experiments is not easy. Usually, we determine the meaning of it by considering the data from the other kinds of experiments. As shown in this manuscript, translocation of β-catenin to cell surface by enforced expression of E-cadherin is used for it (Fig 3C, D). However, nuclear signals appeared in HEK293T cells (Fig 1A. ABC staining) don't indicate the active β-catenin, because there is no transcriptional activity of TCF-dependent reporter construct as shown by the previous work (EMBO report 3, 63–68, 2002). So extensive characterization of monoclonal antibody 8E7 is only useful if there is no way to examine the activity of Wnt signaling, like immunohistochemical study.

From this point of view, the authors show the reasonable imunohistochemical staining data (Figure 4) using 8E7 antibody in the similar manner (using citrate buffer) as previously shown by Dr. Clevers (J Biol Chem 277, 17901). In addition, they estimate their procedure's accuracy by comparing the similar staining patterns observed by Dr. Clevers (J Biol Chem 277, 17901). These facts indicate that the author's information provided in this manuscript is not enough for wide range of readers.

I speculate that what the authors want to say in this manuscript is the signals observed using 8E7 antibody would make some troubles in estimating actual Wnt activity. So I want to know such situations. If the amount is not enough to detect using any experimental procedures, non-specific signals will appear by enhancing the signals. Accordingly, I think that the author's idea will be easily accepted by the readers if the authors will show some examples of the difficulty in discriminating the signals of ABC and those of non-specific. However, the author performed their experiment mainly using L cells. In L cells, the amount of non-phosphorylated β-catenin is very low and slight induction can be seen by Wnt3a expression (Fig 2B, Fig 3B). In addition, the ABC signals observed in L cells is not so strong as that of SW480 cells (Fig 3A), which express much amount of unphosphorylated β-catenin as shown in Fig 3B. Therefore, I suggest that the authors should show additional experimental results using L cells and/or 293T cells stimulated with LiCl (Western blotting and Immunofluorescence staining of these cells using 8E7). The details of staining procedure, magnification of the photo and the exposure time should be shown. If the authors succeed in showing the difficulty of assessing the ABC amount by immunostaining, then the authors need to show the way to overcome this by improving their staining protocol. If the authors successfully show these issues, their results will be very informative for many readers.

### Specific points

Figure 1, 2, 3: It would be better to show the magnification and exposure time.

#### Our response

We firmly agree with the reviewer's acknowledgment that most antibodies cross-react with epitopes present within other proteins. We also agree that we need to establish under what specific conditions this can be a problem. We now show in Figure 1D&1E, immunoblots indicating that the induction of ABC by LiCl or Wnt3a treatment *is similar in abundance to *the non-specific band (*) in HEK cells. While we cannot say that the ~160 kDa species is the cross-reacting nuclear antigen (e.g., the nuclear antigen could be a > 250 kDa protein not detected by our gel/transfer protocols), this blot *does *show that the "signal-to-noise" ratio in Wnt/LiCl-treated HEK cells is low (i.e., ABC: 160 kDa species is 1:1). Unless a cell type has an abundance of ABC relative to this background nuclear antigen (or high "signal to noise" ratio), it will be difficult to know whether a nuclear ABC signal is truly due to ABC, outside of the cadherin-sequestration experiment proposed in Figure 3. At the reviewer's request, we now provide a shorter image exposure time for the ABC antibody that is now matched with the pan β-catenin antibody exposure (Compare Figure 1C with A&B) to show that it is indeed difficult to distinguish true ABC "signal" to background "noise" in the HEK cell system.

#### Review#3 response

At first, I thought that longer exposure might be the cause of nuclear signals appeared in HEK 293 cells without LiCl stimulation, because ABC signals appeared in Figure 1B seemed saturated in comparison with that in Figure 1A. So I have requested the authors to show the images exposed shorter time in addition to western blot analysis. Some images of ABC staining without stimulation showed reduced signals than that with stimulation, as expected (not shown). However, other images actually showed strong nuclear staining without stimulation as shown in Figure 1C. So I understood the existence of strong nuclear signals, which were not ABC, in HEK293 cells using 8E7 antibody.

Furthermore, their data contain more information, which is not addressed so much in the text. Activation of Wnt signal can be estimated by total β-catenin staining in Wnt3a-expressing L cells, but not ABC, because of the weak induction of dephosphorylated β-catenin (Figure 2A, 2B, 3A, 3B). Thus, Wnt activity cannot be estimated in certain cell types using 8E7 antibody. This is an example of the limitation using 8E7 antibody for estimating the Wnt activity. As expected, low signal and high noise is the problematic situation when estimating the genuine signals. As described here, the authors have successfully shown the difficulty using this antibody. One way to look at this finding is that slight induction of ABC is actually exhibited the exact Wnt activity in L cells. But there is another side to this finding. This data may further indicate the existence of unknown regulatory mechanisms of β-catenin stabilization in Wnt-signaling other than inhibition of phosphorylation. Obviously, monoclonal antibody, 8E7 is an important tool for research in the field of Wnt. To understand the relation between biological significance of the weak Wnt activity and ABC signals is one of the important issues to be clarified.

For these reasons, I think that this manuscript is a reasonable paper for publication in Biology Direct.

#### Our response

We agree with Dr. Sugai's suggestion that the Abstract and Conclusions of our manuscript could be written more clearly, so as to explain our findings more succinctly and directly. The following additions have now been incorporated:

##### Abstract

"In cell types commonly used to study Wnt activation, this non-specific nuclear staining can be robust, obscuring the ABC signal. Definitive detection of nuclear localized ABC can be confirmed through an ability of classical cadherins to sequester ABC to cell junctions. In tissues, milder antigen retrieval methods can reduce the accessibility of mAb 8E7 to this cross-reacting nuclear antigen."

##### Conclusion

"These findings demonstrate that the ability of 8E7 to accurately capture evidence of Wnt signaling in cells or tissues critically depends on the strength and/or duration of the Wnt signal, relative to the accessibility of this background antigen."
